# The relative risk of second primary cancers in Queensland, Australia: a retrospective cohort study

**DOI:** 10.1186/1471-2407-11-83

**Published:** 2011-02-23

**Authors:** Danny R Youlden, Peter D Baade

**Affiliations:** 1 Viertel Centre for Research in Cancer Control, Cancer Council Queensland, Brisbane, Australia; 2 School of Public Health, Queensland University of Technology, Brisbane, Australia

## Abstract

**Background:**

Cancer survivors face an increased likelihood of being subsequently diagnosed with another cancer. The aim of this study was to quantify the relative risk of survivors developing a second primary cancer in Queensland, Australia.

**Methods:**

Standardised incidence rates stratified by type of first primary cancer, type of second primary cancer, sex, age at first diagnosis, period of first diagnosis and follow-up interval were calculated for residents of Queensland, Australia, who were diagnosed with a first primary invasive cancer between 1982 and 2001 and survived for a minimum of 2 months.

**Results:**

A total of 23,580 second invasive primary cancers were observed over 1,370,247 years of follow-up among 204,962 cancer patients. Both males (SIR = 1.22; 95% CI = 1.20-1.24) and females (SIR = 1.36; 95% CI = 1.33-1.39) within the study cohort were found to have a significant excess risk of developing a second cancer relative to the incidence of cancer in the general population. The observed number of second primary cancers was also higher than expected within each age group, across all time periods and during each follow-up interval.

**Conclusions:**

The excess risk of developing a second malignancy among cancer survivors can likely be attributed to factors including similar aetiologies, genetics and the effects of treatment, underlining the need for ongoing monitoring of cancer patients to detect subsequent tumours at an early stage. Education campaigns developed specifically for survivors may be required to lessen the prevalence of known cancer risk factors.

## Background

The number of people being diagnosed with cancer worldwide is rising sharply, mainly as a result of population growth, ageing and increases in the prevalence of several lifestyle factors related to cancer risk [1,2]. Combined with improvements in survival, due in part to both earlier detection and better treatment, more people are living with a diagnosis of cancer than ever before, and this increasing trend is likely to continue [3].

One of the consequences of surviving cancer is the increased likelihood of being diagnosed with a second primary cancer. Among other things, these second cancers may be the result of lifestyle choices, genetics, environmental exposures (all of which may also be related to the first cancer) and late effects of treatment [3,4].

For the patient and their treating clinician, quantifying and characterising the risks for second malignancies has important implications for screening (particularly when effective screening methods such as mammography are available), prevention strategies and counselling [3,5,6]. Identifying cancers which have an elevated likelihood of occurring together is also a useful starting point for investigating possible shared aetiologies and mechanisms of carcinogenesis [7].

This study reports for the first time the relative risks of a second cancer for people diagnosed with a first primary cancer in Queensland, Australia.

## Methods

A retrospective cohort design was used for this analysis. De-identified case records were obtained from the Queensland Cancer Registry (QCR), a population-based registry covering the entire state. All public and private hospitals, nursing homes and pathology services throughout Queensland are required by law to notify the QCR about any patients diagnosed with cancer, except for non-melanoma skin cancers [8].

The study cohort included all Queensland residents diagnosed with a first primary invasive cancer between 1982 and 2001 who survived for a minimum of 2 months. Since classifications for childhood cancers are different to adult cancers [9], we decided to restrict the cohort to people who were 15 years or older at the time of first diagnosis. A small number of records were excluded because information was missing for age (n = 20), or because the person was known to have had a first primary cancer diagnosed prior to 1982 (n = 76).

The cohort was followed up until 31^st ^December 2006 (allowing a potential minimum of five years and a maximum of 25 years after the initial diagnosis) to ascertain the occurrence of second primary invasive cancers. Histologically similar cases of cancer at the same body site were included, unless the medical record indicated that the tumour was recurrent or metastatic. Synchronous primary cancers (those diagnosed within 2 months of the first primary cancer) [10] were excluded because they were more likely to have been diagnosed as a result of detection bias [6]. Third or subsequent primary cancers were not considered in this analysis.

Cancers of various body sites were analysed in a separate group if they averaged more than 100 eligible first primary cancers per year. These cancers are shown in Table 1. Cancers of the head and neck (ICD-O-3 topography codes C00-C14 and C30-C32) were grouped together prior to analysis, as were cancers of the colon and rectum (C18-C20 and C218). All remaining types of cancer were analysed collectively in the category labelled "Other", including cases where site was ill-defined or unknown.

**Table 1:** Characteristics of the study cohort

	First Primary Cancers		Second Primary Cancers	
	
	Number of cases	%	Number of cases	%
**Total**	204,962	100.0	23,580	100.0

***Sex***				
Males	110,961	54.1	14,388	61.0
Females	94,001	45.9	9,192	39.0
***Age at first diagnosis***				
15-49 years	41,690	20.3	3,150	13.4
50-64 years	60,573	29.6	7,687	32.6
65 years and over	102,699	50.1	12,743	54.0
***Period of first diagnosis***				
1982-1986	36,216	17.7	4,716	20.0
1987-1991	43,904	21.4	5,841	24.8
1992-1996	57,381	28.0	6,972	29.6
1997-2001	67,461	32.9	6,051	25.7
***Follow-up interval***				
2 months to less than 1 year	n.a.		2,511	10.6
1 year to less than 5 years	n.a.		8,924	37.8
5 years to less than 10 years	n.a.		7,286	30.9
10 years or longer	n.a.		4,859	20.6
***Type of first primary cancer***				
Head and neck (C00-C14,C30-C32)	10,942	5.3	1,989	8.4
Oesophagus (C15)	2,075	1.0	105	0.4
Stomach (C16)	4,103	2.0	206	0.9
Colorectal (C18-C20,C218)	27,814	13.6	3,046	12.9
Pancreas (C25)	2,817	1.4	49	0.2
Lung (C33-C34)	17,347	8.5	729	3.1
Melanoma (C44,M872-M879)	29,289	14.3	5,092	21.6
Breast - female (C50)	26,725	13.0	2,962	12.6
Cervix (C53)	3,492	1.7	300	1.3
Uterus (C54)	3,527	1.7	409	1.7
Ovary (C56)	2,864	1.4	164	0.7
Prostate (C61)	23,122	11.3	3,006	12.7
Kidney (C64-C66,C68)	4,927	2.4	655	2.8
Bladder (C67)	8,719	4.3	1,680	7.1
Brain & CNS (C70-C72)	2,566	1.3	50	0.2
Thyroid (C73)	2,302	1.1	215	0.9
Non-Hodgkin lymphoma (M967-M972)	6,618	3.2	689	2.9
Lymphoid leukaemia (M982-M983)	2,645	1.3	422	1.8
Myeloid leukaemia (M984-M993)	2,195	1.1	125	0.5
Myeloma/PCT (M973)	2,174	1.1	134	0.6
Other	18,699	9.1	1,553	6.6

n.a. = not applicable; CNS = central nervous system; PCT = plasma cell tumours.

Person years at risk (PYAR) among people diagnosed with a first primary cancer was calculated as the time from 2 months after diagnosis until 31 December 2006, date of death or date of diagnosis of a second primary cancer, whichever came first. Data were stratified by type of first primary cancer, type of second primary cancer, sex, age at first diagnosis, period of first diagnosis and follow-up interval. Analysis by period of first diagnosis was restricted to five years of follow-up to allow more consistent comparisons across time. The expected number of second primary cancers in each stratum was calculated by multiplying the sum of PYAR by the cancer-specific incidence rate experienced by the general Queensland population, matched by sex, age group and time period. Standardised incidence ratios (SIRs) were then obtained by dividing the observed number of cases of second primary cancer by the expected number. The SIR is thus used to estimate the risk of a cancer patient developing a second primary malignancy relative to the incidence of cancer among the general population. Confidence intervals (CIs) for the SIRs were derived from the Poisson distribution [11] and calculated at the 95% level of certainty.

All analyses were conducted using SAS v9.2 for Windows. Data required for this study was non-identifiable so no ethics committee approval was necessary.

## Results

The basic characteristics of the study cohort are summarised in Table 1. Among the 204,962 eligible cancer patients, a total of 23,580 second invasive primary cancers were observed during 1,370,247 years of follow-up (median follow-up = 5.5 years per person, interquartile range = 1.3 to 10.2 years per person). In terms of absolute numbers, second primary cancers were more common among males and increased with older age, which is consistent with the distribution of first primary cancers. About one in ten (10.6%) of the second primary cancers were diagnosed within a year of the first diagnosis, while more than one in five (20.6%) were diagnosed at least 10 years afterwards. The highest proportions of second primary cancers occurred following an initial diagnosis of melanoma (21.6%), colorectal cancer (12.9%), prostate cancer (12.7%), or female breast cancer (12.6%).

### Relative risk of second primary cancers by sex

Compared to the incidence of cancer in the general Queensland population, both males (SIR = 1.22; 95% CI = 1.20-1.24) and females (SIR = 1.36; 95% CI = 1.33-1.39) in the study cohort exhibited an increased risk of developing a second primary cancer (Table 2). Significantly increased relative risks of invasive cancer were recorded among males following diagnosis of head and neck cancer, oesophageal cancer, lung cancer, melanoma, kidney cancer, bladder cancer, thyroid cancer, non-Hodgkin lymphoma, lymphoid leukaemia or myeloid leukaemia. In contrast, males initially diagnosed with either prostate or stomach cancer subsequently experienced a significantly lower risk of cancer compared to the general population.

**Table 2:** Relative risk of second primary cancer by type of first primary cancer and sex, Queensland, 1982-2006

	Sex			
	
	Males		Females	
**First primary cancer**	**Obs**.	**SIR** **(95% CI)**	**Obs**.	**SIR ** **(95% CI)**

Head and neck	1,620	**1.70** **(1.62-1.79)**	369	**1.84** **(1.66-2.04)**

Oesophagus	78	**1.43** **(1.13-1.79)**	27	1.06 (0.70-1.54)

Stomach	150	***0.83*** ***(0.70-0.98)***	56	1.00 (0.75-1.30)

Colorectal	1,899	1.04 (0.99-1.08)	1,147	**1.08** **(1.02-1.15)**

Pancreas	31	0.92 (0.63-1.31)	18	0.85 (0.50-1.34)

Lung	552	**1.18** **(1.08-1.28)**	177	**1.41** **(1.21-1.64)**

Melanoma	3,267	**1.77** **(1.71-1.83)**	1,825	**1.70** **(1.62-1.78)**

Breast - female	n.a.	n.a.	2,962	**1.31** **(1.27-1.36)**

Cervix	n.a.	n.a.	300	**1.36** **(1.21-1.52)**

Uterus	n.a.	n.a.	409	**1.16** **(1.05-1.28)**

Ovary	n.a.	n.a.	164	1.12 (0.95-1.30)

Prostate	3,006	***0.83*** ***(0.80-0.86)***	n.a.	n.a.

Kidney	434	**1.39** **(1.27-1.53)**	221	**1.69** **(1.47-1.93)**

Bladder	1,370	**1.40** **(1.33-1.48)**	310	**1.61** **(1.43-1.79)**

Brain & CNS	31	1.05 (0.71-1.49)	19	0.94 (0.57-1.47)

Thyroid	70	**1.47** **(1.15-1.86)**	145	**1.35** **(1.14-1.59)**

Non-Hodgkin lymphoma	411	**1.37** **(1.24-1.51)**	278	**1.36** **(1.20-1.53)**

Lymphoid leukaemia	297	**1.66** **(1.48-1.86)**	125	**1.40** **(1.17-1.67)**

Myeloid leukaemia	87	**1.44** **(1.15-1.77)**	38	**1.25** **(0.88-1.71)**

Myeloma/PCT	93	1.04 (0.84-1.27)	41	0.89 (0.64-1.21)

Other	992	**1.23** **(1.16-1.31)**	561	**1.37** **(1.26-1.49)**

All cancers combined	14,388	**1.22** **(1.20-1.24)**	9,192	**1.36** **(1.33-1.39)**

Obs. = Observed number of second primary cancers; SIR = standardised incidence ratio; CI = confidence interval; n.a. = not applicable; CNS = central nervous system; PCT = plasma cell tumours. SIRs shown in normal bold font indicate significantly increased risk; SIRs shown in bold italics indicate significantly decreased risk

Within the female cohort, the relative risk of a second cancer was higher for those diagnosed with head and neck cancer, colorectal cancer, lung cancer, melanoma, breast cancer, cervical cancer, uterine cancer, kidney cancer, bladder cancer, thyroid cancer, non-Hodgkin lymphoma, lymphoid leukaemia or myeloid leukaemia. There were no types of cancer for which female survivors had a significantly lower risk of developing a second invasive cancer in relation to the general population.

### Relative risk of second primary cancers by age group at diagnosis

The risk of a second malignancy was higher in comparison to the general population for each of the three age groups for all first primary cancers combined, but tended to decrease as age at first diagnosis increased (Table 3) - 15-49 years (SIR = 1.84; 95% CI = 1.77-1.90), 50-64 years (SIR = 1.39; 95% CI = 1.36-1.42) and 65 years and older (SIR = 1.23; 95% CI = 1.20-1.25). Different patterns emerged within the various cancer-specific cohorts. An elevated relative risk across all three age groups was found following head and neck cancer, lung cancer, melanoma, female breast cancer, cervical cancer, kidney cancer, bladder cancer, non-Hodgkin lymphoma and lymphoid leukaemia. For oesophageal and colorectal cancer, significantly increased relative risks were only observed among those aged under 65 years at first diagnosis, while for pancreatic and brain cancers the risk was elevated in the younger age groups but was lower than expected for people aged 65 years and over. Consistently decreased relative risks were recorded within each age group for males with prostate cancer, although the SIR was not statistically significant for those aged 15-49 years.

**Table 3:** Relative risk of second primary cancer by type of first primary cancer and age group at first diagnosis, Queensland, 1982-2006

	Age group at first diagnosis					
	
	15-49 years		50-64 years		65 years and over	
**First primary cancer**	**Obs**.	**SIR** ** (95% CI)**	**Obs**.	**SIR** ** (95% CI)**	**Obs**.	**SIR** ** (95% CI)**

Head and neck	223	**2.07** **(1.80-2.36)**	884	**2.20** **(2.06-2.35)**	882	**1.77** **(1.65-1.89)**

Oesophagus	6	**4.00** **(1.47-8.70)**	39	**1.70** **(1.21-2.32)**	60	1.16 (0.88-1.49)

Stomach	17	1.59 (0.93-2.55)	60	1.04 (0.79-1.34)	129	0.91 (0.76-1.08)

Colorectal	188	**1.58** **(1.36-1.82)**	994	**1.15** **(1.08-1.22)**	1,864	1.03 (0.99-1.08)

Pancreas	--	--	26	**1.60** **(1.05-2.35)**	23	***0.61*** ***(0.39-0.92)***

Lung	52	**2.46** **(1.84-3.23)**	270	**1.55** **(1.37-1.74)**	407	**1.24** **(1.12-1.36)**

Melanoma	1,121	**2.05** **(1.94-2.18)**	1,755	**1.80** **(1.72-1.89)**	2,216	**1.74** **(1.67-1.81)**

Breast - female	689	**1.86** **(1.72-2.00)**	1,047	**1.29** **(1.21-1.37)**	1,226	**1.15** **(1.08-1.21)**

Cervix	129	**1.33** **(1.11-1.57)**	94	**1.49** **(1.21-1.83)**	77	**1.27** **(1.01-1.59)**

Uterus	47	**1.63** **(1.20-2.17)**	164	1.07 (0.91-1.25)	198	**1.16** **(1.00-1.33)**

Ovary	33	1.25 (0.86-1.76)	64	1.12 (0.87-1.44)	67	1.06 (0.82-1.34)

Prostate	7	0.81 (0.32-1.66)	554	***0.87*** ***(0.80-0.95)***	2,445	***0.82*** ***(0.79-0.85)***

Kidney	52	**1.63** **(1.22-2.14)**	230	**1.59** **(1.39-1.81)**	373	**1.57** **(1.42-1.74)**

Bladder	81	**2.02** **(1.60-2.51)**	465	**1.63** **(1.49-1.79)**	1,134	**1.70** **(1.60-1.80)**

Brain & CNS	27	**1.64** **(1.08-2.39)**	15	0.94 (0.52-1.55)	8	***0.48*** ***(0.21-0.95)***

Thyroid	65	1.22 (0.94-1.55)	84	**1.39** **(1.11-1.72)**	66	**1.37** **(1.06-1.74)**

Non-Hodgkin lymphoma	81	**1.60** **(1.27-1.99)**	261	**1.57** **(1.39-1.77)**	347	**1.23** **(1.10-1.36)**

Lymphoid leukaemia	27	**2.68** **(1.77-3.90)**	131	**1.69** **(1.42-2.01)**	264	**1.57** **(1.39-1.77)**

Myeloid leukaemia	22	**2.23** **(1.40-3.38)**	33	1.39 (0.96-1.95)	70	**1.35** **(1.05-1.70)**

Myeloma/PCT	8	1.40 (0.61-2.77)	47	1.35 (0.99-1.79)	79	0.89 (0.70-1.10)

Other	273	**1.69** **(1.49-1.90)**	472	**1.43** **(1.30-1.56)**	808	**1.20** **(1.12-1.29)**

All cancers combined	3,150	**1.84** **(1.77-1.90)**	7,687	**1.39** **(1.36-1.42)**	12,743	**1.23** **(1.20-1.25)**

Obs. = Observed number of second primary cancers; SIR = standardised incidence ratio; CI = confidence interval; CNS = central nervous system; PCT = plasma cell tumours. SIRs shown in normal bold font indicate significantly increased risk; SIRs shown in bold italics indicate significantly decreased risk. SIRs for pancreatic cancer for the age groups 15-49 years and 50-64 years have been combined due to less than 5 observed cases aged 15-49 years.

### Relative risk of second primary cancers by time period of first diagnosis

There was some evidence that the more recently a first primary cancer was diagnosed the higher the relative risk of a second primary cancer, with a gradual increase across the four time periods for all cancers combined (Table 4): 1982-1986 (SIR = 1.14; 95% CI = 1.08-1.20), 1987-1991 (SIR = 1.22; 95% CI = 1.17-1.28), 1992-1996 (SIR = 1.36; 95% CI = 1.31-1.41) and 1997-2001 (SIR = 1.46; 95% CI = 1.41-1.50). In particular, the SIRs for people with colorectal cancer, lung cancer, breast cancer, thyroid cancer, non-Hodgkin lymphoma, lymphoid leukaemia and myeloid leukaemia were only significant for the later time periods. Among men with prostate cancer, the risk of developing a second primary cancer was lower than the incidence of cancer experienced by males in the general population irrespective of the year of first diagnosis, except for the latest period (1997-2001). Several cancer-specific cohorts had SIRs that were significantly higher than expected in each time period, including head and neck cancer, melanoma, kidney cancer and bladder cancer.

**Table 4:** Relative risk of second primary cancer by type of first primary cancer and time period of first diagnosis, Queensland, 1982-2006

	Time period of first diagnosis							
	
	1982-1986		1987-1991		1992-1996		1997-2001	
**First primary cancer**	**Obs**.	**SIR ** **(95% CI)**	**Obs**.	**SIR ** **(95% CI)**	**Obs**.	**SIR ** **(95% CI)**	**Obs**.	**SIR** ** (95% CI)**

Head and neck	165	**1.92** **(1.64-2.23)**	204	**1.88** **(1.63-2.16)**	271	**1.98** **(1.75-2.23)**	255	**1.93** **(1.70-2.18)**

Oesophagus	8	1.53 (0.66-3.02)	10	0.90 (0.43-1.66)	23	**1.60** **(1.01-2.39)**	23	1.19 (0.76-1.79)

Stomach	18	0.75 (0.44-1.18)	27	1.02 (0.67-1.49)	30	0.91 (0.62-1.31)	36	1.09 (0.76-1.51)

Colorectal	198	0.89 (0.77-1.02)	273	0.95 (0.84-1.07)	411	1.07 (0.97-1.18)	559	**1.20** **(1.11-1.31)**

Pancreas	7	1.20 (0.48-2.47)	5	0.61 (0.20-1.42)	10	0.98 (0.47-1.81)	12	0.83 (0.43-1.45)

Lung	71	1.06 (0.83-1.34)	93	1.14 (0.92-1.40)	147	**1.61** **(1.36-1.89)**	148	**1.30** **(1.10-1.53)**

Melanoma	311	**1.81** **(1.62-2.02)**	471	**1.89** **(1.72-2.07)**	639	**2.04** **(1.88-2.20)**	835	**2.02** **(1.89-2.16)**

Breast - female	152	0.97 (0.82-1.14)	199	0.94 (0.81-1.08)	359	**1.32** **(1.19-1.47)**	504	**1.37** **(1.25-1.50)**

Cervix	24	1.29 (0.83-1.92)	38	**1.88** **(1.33-2.58)**	34	**1.64** **(1.14-2.29)**	26	**1.55** **(1.01-2.27)**

Uterus	30	1.14 (0.77-1.63)	35	1.18 (0.82-1.64)	35	0.87 (0.60-1.21)	63	1.30 (0.99-1.66)

Ovary	16	1.36 (0.78-2.20)	10	0.64 (0.31-1.18)	26	1.35 (0.88-1.98)	24	1.02 (0.66-1.52)

Prostate	163	***0.64*** ***(0.54-0.74)***	291	***0.76*** ***(0.68-0.85)***	603	***0.84*** ***(0.77-0.91)***	655	0.95 (0.88-1.02)

Kidney	44	**1.48** **(1.08-1.99)**	73	**1.79** **(1.40-2.25)**	92	**1.53** **(1.24-1.88)**	160	**2.04** **(1.74-2.38)**

Bladder	137	**1.46** **(1.22-1.72)**	189	**1.78** **(1.53-2.05)**	238	**1.84** **(1.61-2.09)**	353	**2.22** **(2.00-2.47)**

Brain & CNS	--	--	5	1.00 (0.33-2.34)	--	--	9	1.10 (0.50-2.09)

Thyroid	9	1.25 (0.57-2.38)	12	1.25 (0.64-2.18)	29	**1.64** **(1.10-2.36)**	46	**1.67** **(1.22-2.23)**

Non-Hodgkin lymphoma	42	1.09 (0.79-1.48)	62	1.15 (0.88-1.48)	88	**1.25** **(1.00-1.54)**	144	**1.49** **(1.25-1.75)**

Lymphoid leukaemia	24	1.00 (0.64-1.48)	33	1.25 (0.86-1.75)	63	**1.68** **(1.29-2.15)**	88	**1.71** **(1.37-2.10)**

Myeloid leukaemia	13	1.23 (0.66-2.11)	11	0.91 (0.45-1.62)	26	1.52 (0.99-2.22)	34	**2.05** **(1.42-2.86)**

Myeloma/PCT	21	1.37 (0.85-2.10)	7	***0.46*** ***(0.19-0.95)***	19	0.83 (0.50-1.29)	39	1.05 (0.75-1.44)

Other	79	0.94 (0.74-1.17)	136	**1.22** **(1.03-1.45)**	241	**1.43** **(1.26-1.62)**	318	**1.43** **(1.28-1.60)**

All cancers combined	1,534	**1.14** **(1.08-1.20)**	2,184	**1.22** **(1.17-1.28)**	3,386	**1.36** **(1.31-1.41)**	4,331	**1.46** **(1.41-1.50)**

Obs. = Observed number of second primary cancers; SIR = standardised incidence ratio; CI = confidence interval; CNS = central nervous system; PCT = plasma cell tumours. Analysis by period of first diagnosis was restricted to five years of follow-up to allow more consistent comparisons across time. SIRs shown in normal bold font indicate significantly increased risk; SIRs shown in bold italics indicate significantly decreased risk. Estimates are not shown for Brain & CNS for the periods 1982-1986 and 1992-1996 due to observed cell counts of less than 5.

### Relative risk of second primary cancers by follow-up interval

The risk of a second primary cancer for all survivors combined remained consistently elevated compared to the general population during each follow-up interval (Table 5): 2 months to less than 1 year after first diagnosis (SIR = 1.32; 95% CI = 1.27-1.37), 1 year to less than 5 years (SIR = 1.33; 95% CI = 1.31-1.36), 5 years to less than 10 years (SIR = 1.39; 95% CI = 1.35-1.42) and 10 years or longer (SIR = 1.28; 95% CI = 1.24-1.31). A significantly increased relative risk across all follow-up intervals was also observed following a diagnosis of head and neck cancer, melanoma, kidney cancer, bladder cancer or lymphoid leukaemia, while the risks were significantly higher from 1 year or longer after diagnosis for colorectal cancer, lung cancer, female breast cancer or non-Hodgkin lymphoma. The relative risk of developing another cancer was significantly higher among cervical cancer survivors up to 10 years after the initial diagnosis but not after that. Prostate cancer patients were found to have subsequent risks that remained lower than the matching population, particularly 10 or more years after initial diagnosis.

**Table 5:** Relative risk of second primary cancer by type of first primary cancer and follow-up interval, Queensland, 1982-2006

	Follow-up interval							
	
	2 months to less than 1 year		1 year to less than 5 years		5 years to less than 10 years		10 years or longer	
**First primary cancer**	**Obs**.	**SIR** ** (95% CI)**	**Obs**.	**SIR** ** (95% CI)**	**Obs**.	**SIR** ** (95% CI)**	**Obs**.	**SIR** ** (95% CI)**

Head and neck	164	**1.67** **(1.42-1.95)**	731	**2.00** **(1.86-2.15)**	651	**2.10** **(1.94-2.26)**	443	**1.89** **(1.72-2.08)**

Oesophagus	22	1.22 (0.77-1.85)	42	1.31 (0.95-1.77)	32	**1.76** **(1.20-2.48)**	9	1.11 (0.51-2.10)

Stomach	36	0.97 (0.68-1.35)	75	0.95 (0.74-1.18)	55	1.04 (0.78-1.35)	40	0.99 (0.71-1.35)

Colorectal	310	1.03 (0.92-1.16)	1,131	**1.07** **(1.01-1.13)**	912	**1.11** **(1.04-1.18)**	693	**1.14** **(1.06-1.23)**

Pancreas	14	0.74 (0.40-1.24)	20	1.01 (0.62-1.57)	15	0.99 (0.55-1.64)	--	--

Lung	147	1.06 (0.89-1.24)	312	**1.46** **(1.30-1.63)**	183	**1.71** **(1.47-1.98)**	87	**1.35** **(1.08-1.67)**

Melanoma	458	**2.15** **(1.95-2.35)**	1,798	**1.92** **(1.84-2.02)**	1,553	**1.80** **(1.71-1.89)**	1,283	**1.63** **(1.55-1.73)**

Breast - female	194	1.02 (0.88-1.18)	1,020	**1.25** **(1.17-1.33)**	1,019	**1.44** **(1.35-1.53)**	729	**1.36** **(1.26-1.46)**

Cervix	33	**2.13** **(1.46-2.99)**	89	**1.46** **(1.18-1.80)**	89	**1.43** **(1.15-1.76)**	89	1.09 (0.87-1.34)

Uterus	29	1.03 (0.69-1.48)	134	1.15 (0.96-1.36)	147	**1.36** **(1.15-1.60)**	99	1.00 (0.81-1.21)

Ovary	22	1.22 (0.77-1.85)	54	1.04 (0.78-1.35)	46	1.18 (0.86-1.57)	42	1.11 (0.80-1.50)

Prostate	344	***0.81*** ***(0.73-0.90)***	1,368	***0.84*** ***(0.80-0.89)***	988	***0.88*** ***(0.82-0.93)***	306	***0.66*** ***(0.59-0.74)***

Kidney	115	**2.45** **(2.02-2.94)**	254	**1.57** **(1.38-1.77)**	177	**1.42** **(1.22-1.65)**	109	**1.35** **(1.11-1.63)**

Bladder	249	**2.40** **(2.11-2.72)**	668	**1.73** **(1.61-1.87)**	477	**1.61** **(1.47-1.76)**	286	**1.39** **(1.23-1.56)**

Brain & CNS	6	0.60 (0.22-1.31)	12	0.77 (0.40-1.34)	18	1.44 (0.85-2.28)	14	1.28 (0.70-2.15)

Thyroid	18	1.65 (0.98-2.61)	78	**1.53** **(1.21-1.91)**	63	1.21 (0.93-1.55)	56	1.16 (0.87-1.50)

Non-Hodgkin lymphoma	74	1.23 (0.97-1.55)	262	**1.31** **(1.16-1.48)**	209	**1.46** **(1.27-1.67)**	144	**1.49** **(1.26-1.76)**

Lymphoid leukaemia	52	**1.82** **(1.36-2.39)**	156	**1.40** **(1.19-1.64)**	143	**1.89** **(1.59-2.23)**	71	**1.78** **(1.37-2.22)**

Myeloid leukaemia	27	**1.59** **(1.05-2.31)**	57	**1.44** **(1.09-1.87)**	28	1.40 (0.93-2.03)	13	1.41 (0.75-2.41)

Myeloma/PCT	18	0.76 (0.45-1.21)	68	1.02 (0.79-1.29)	36	1.26 (0.88-1.74)	12	1.12 (0.58-1.95)

Other	179	**1.22** **(1.05-1.42)**	595	**1.35** **(1.25-1.47)**	447	**1.36** **(1.24-1.49)**	332	**1.33** **(1.19-1.48)**

All cancers combined	2,511	**1.32** **(1.27-1.37)**	8,924	**1.33** **(1.31-1.36)**	7,286	**1.39** **(1.35-1.42)**	4,859	**1.28** **(1.24-1.31)**

Obs. = Observed number of second primary cancers; SIR = standardised incidence ratio; CI = confidence interval; CNS = central nervous system; PCT = plasma cell tumours. SIRs shown in normal bold font indicate significantly increased risk; SIRs shown in bold italics indicate significantly decreased risk. SIRs for pancreatic cancer for 5 years to less than 10 years after first diagnosis and 10 years or longer after first diagnosis have been combined due to less than 5 observed cases 10 years or longer after first diagnosis.

### Relative risk by type of first and second primary cancers and sex

**Figure 1:**
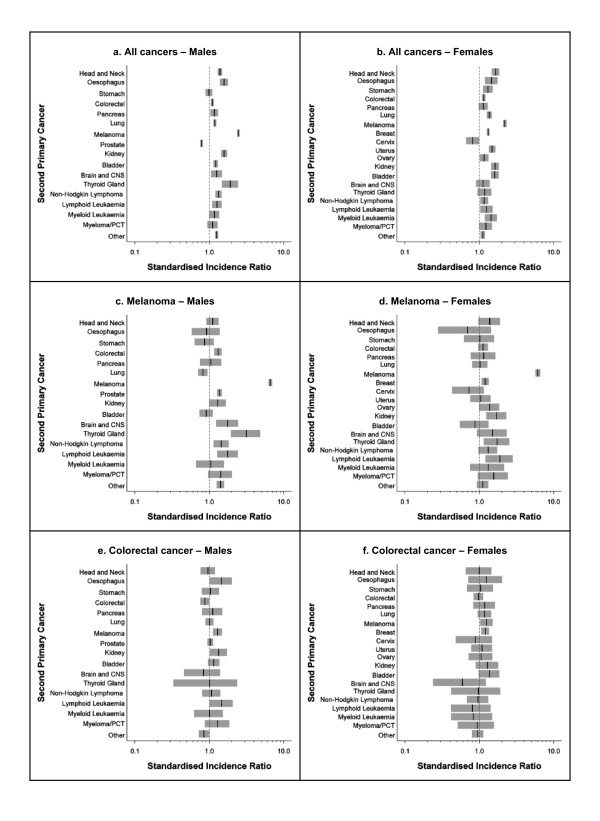
**Relative risk following all cancers combined, melanoma or colorectal cancer by type of second primary cancer and sex, Queensland, 1982-2006**. CNS: central nervous system; PCT: plasma cell tumour.Vertical black line indicates SIR point estimate; grey shading indicates SIR 95% confidence interval. X-axes are shown on a log scale.

**Figure 2:**
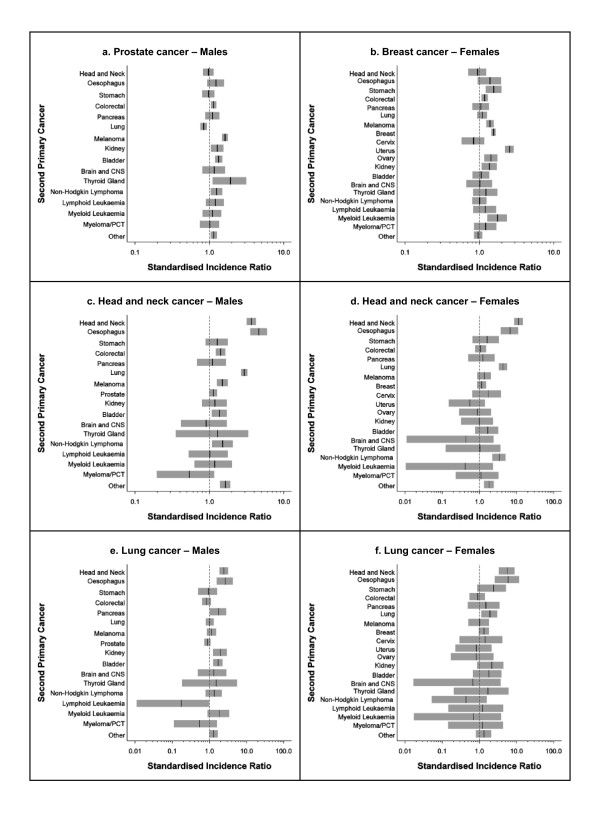
**Relative risk following prostate cancer, breast cancer, head and neck cancer or lung cancer by type of second primary cancer and sex, Queensland, 1982-2006**. CNS: central nervous system; PCT: plasma cell tumour. Vertical black line indicates SIR point estimate; grey shading indicates SIR 95% confidence interval. The SIR for a second primary prostate cancer following a first primary prostate cancer was 0.3 and is not shown in Figure 2a. No cases of lymphoid leukaemia following head and neck cancer were recorded for females (Figure 2d). X-axes are shown on a log scale. Note different scale endpoints for x-axis used in Figures 2d, 2e and 2f.

The relative risks for specific second primary cancers varied substantially according to the type of first primary cancer (Figure 1 and 2). Within the melanoma cohort (Figure 1c and 1d), both males and females were over six times more likely to be diagnosed with another primary melanoma compared to the general population. They also had significantly increased relative risks for several other cancers, including thyroid cancer and lymphoid leukaemia (both males and females), brain cancer, non-Hodgkin lymphoma, prostate cancer and colorectal cancer (males only) and kidney cancer and breast cancer (females only). However, lung cancers occurred less often than expected among males with a first primary melanoma.

An elevated relative risk of melanoma was also observed for both sexes in the colorectal cancer cohort (Figure 1e and 1f). In addition, male colorectal cancer survivors had higher risks for lymphoid leukaemia, oesophageal cancer and kidney cancer but a lower risk of developing a second primary colorectal cancer compared to the general population, while females with colorectal cancer experienced a subsequent increased risk of breast cancer.

Men diagnosed with prostate cancer had significantly increased relative risks for thyroid cancer, melanoma, bladder cancer, kidney cancer, non-Hodgkin lymphoma and colorectal cancer but a significantly decreased risk of lung cancer (Figure 2a). Instances of primary prostate cancer occurring twice in the same person were rare. Female breast cancer survivors were more likely to be diagnosed with uterine cancer, myeloid leukaemia, stomach cancer, breast cancer, ovarian cancer, melanoma, kidney cancer or colorectal cancer than were the general population (Figure 2b).

People initially diagnosed with head and neck cancer were found to have elevated relative risks for a second cancer of the head and neck, oesophageal cancer, lung cancer and non-Hodgkin lymphoma (Figure 2c and 2d). Males with head and neck cancer were also at increased risk of developing melanoma, colorectal cancer or bladder cancer. All lung cancer patients had a significantly increased risk of oesophageal and head and neck cancers compared to other residents of Queensland (Figure 2e and 2f), while males with lung cancer experienced high relative risks of being subsequently diagnosed with kidney, pancreas or bladder cancers and females with lung cancer had a significantly increased risk of developing a second primary lung cancer. Lymphoid leukaemia occurred less often among males following lung cancer than in the general population, although both the observed and expected number of cases were small.

## Discussion

Cancer patients in our study cohort were at significantly higher risk of a second diagnosis compared to the underlying incidence rates experienced in the entire population of Queensland. Although there was some variation in the size of the estimated relative risks, a consistent pattern of increased risk following all first primary cancers combined was seen for both males and females, and across all age groups at first diagnosis, time periods of first diagnosis and follow up intervals.

Our data offers further evidence of significant associations between particular types of first and second primary cancers that have been previously documented elsewhere in Australia and around the world [6,12-16]. Some examples include the mutual increased risks between head and neck, lung and oesophageal cancers and the relationship between melanoma and both prostate and female breast cancers.

Much of the elevated risk of being diagnosed with a second malignancy can be attributed to risk behaviours (such as smoking, harmful levels of alcohol consumption and poor diet), inherited susceptibilities and/or the medical treatment that cancer survivors have received [3,4,6]. On occasions, treatment can also have the opposite effect of reducing the risk of subsequent diagnosis. As was noted in another recent study of second primary cancer [13], the overall lower SIRs observed among prostate cancer survivors are mainly due to extremely low rates of reoccurrence as the organ is often completely removed as part of treatment. Given that prostate cancer is the most common cancer diagnosed for males in Queensland, this also helps to explain why the total relative risk is lower for male survivors compared to female survivors.

Another potential explanation for the difference in risk between cancer survivors and the general population is that some demographic factors may not be comparable between the two groups. Although the relative risks were based on calculations matched by sex, age group and time period, it is possible that other qualities, such as socioeconomic status, may vary between people who have been diagnosed with cancer and those who have not. This is likely to be the reason for at least part of the reduced risk of lung cancer (which has higher incidence among lower socioeconomic groups) for males following melanoma (which is more common in segments of the community with higher socioeconomic status) [6,17]. A similar explanation could be used for the deficit of lung cancer after a diagnosis of prostate cancer. In contrast, the high reciprocal relative risks of melanoma with both female breast cancer and prostate cancer may correlate with the higher incidence for each of these malignancies among more affluent populations [18-20].

One of the interesting relationships that emerged from the analysis was the variation in relative risk of second primary cancer by age at first diagnosis following cancer of the brain and central nervous system. Younger survivors (15-49 years old) had an increased risk of being diagnosed with a second primary cancer while survivors aged 65 years or over had a decreased risk. This is most likely due to the various histopathological subtypes of brain tumours which are more common in the different age groups. For example, glioblastoma tends to be diagnosed at an older age and is associated with poor survival, allowing a limited time for treatment-related second primary cancers to appear [6].

The reasons behind an increased relative risk of second primary cancer following certain types of cancer among survivors who were diagnosed more recently are unknown. Tsukuma *et al. *[16] described a similar pattern in Japan, and suggested that apparent increases in risk may be due to improved follow-up and surveillance of cancer patients. Another possible cause is the change in treatment modalities over time [4,21].

Second primary cancers that arise due to the effects of treatment for the initial cancer might be expected to occur many years after the first diagnosis. However, the increase in relative risk remained fairly consistent irrespective of time since diagnosis, in accordance with results published elsewhere in Australia [13] and the United States [6].

The main strengths of this study include the extensive population-based coverage achieved by the QCR for the reporting of cancers among Queensland residents, combined with a high level of histological verification (88% in 2006) [8], which is important for distinguishing between new primary cancers and metastases of an existing cancer. Since all data used in this study have been collected prospectively for administrative purposes, and coded independently of the hypotheses, the opportunity for recall or information bias has been removed.

Increased medical surveillance of newly-diagnosed cancer patients may introduce a detection bias for second primary cancers. The likelihood of this happening has been reduced by only considering metachronous primary cancers, with a two-month window between first and second diagnosis. It is also possible that some second primary cancers were incorrectly classified as first primary cancers, especially for cancers diagnosed soon after the establishment of the QCR in the early 1980s; we are unable to quantify the impact of this on the observed results.

While acknowledging that the study cohort and comparison population were not independent, with the population containing people already diagnosed with cancer, this proportion was less than 0.5% in any given year. Finally, as a result of the large number of comparisons made, the possibility that some of the SIR estimates have been spuriously identified as statistically significant needs to be considered, particularly those based on small numbers of primary or secondary cancers, and these results should therefore be interpreted with due caution.

## Conclusions

Our results demonstrate that cancer survivors in Queensland, Australia, like those in other countries, are confronted by a very real, ongoing risk of developing a second primary cancer that is significantly higher than the incidence of cancer experienced by the general population. Some first and second primary cancers share common aetiologies, making it imperative that cancer patients adopt a healthier lifestyle in order to lessen their chances of subsequent diagnoses [4,22]. Recent studies have reported little difference in the health behaviours of cancer survivors compared to the wider community [23-25], suggesting that health promotion efforts may need to be specifically targeted at people diagnosed with cancer due to their subsequent increased risk. Further work is needed to determine exactly how beneficial changes to lifestyle may be in regard to the risk of developing a second primary cancer [26]. Even so, some second primary cancers will be unavoidable, and as the number of cancer survivors continues to grow, the importance of ongoing medical supervision and screening to detect second primary cancers at an earlier stage and thereby improve the effectiveness of treatment will remain critical.

## Competing interests

The authors declare that they have no competing interests.

## Authors' contributions

DY conducted the statistical analysis and drafted the manuscript. PB conceived the study and edited the draft manuscript. Both authors read and approved the final manuscript.

## Pre-publication history

The pre-publication history for this paper can be accessed here:

http://www.biomedcentral.com/1471-2407/11/83/prepub
